# Supplementary material for: Community deployment of metofluthrin emanators to control indoor Aedes aegypti: Efficacy results from a crossover trial in Yucatan, Mexico
Source: PLoS Negl Trop Dis. 2025 Sep 5;19(9):e0012883. doi: 10.1371/journal.pntd.0012883 (PMC12422581; doi:10.1371/journal.pntd.0012883)
Supplement: S1 Fig — (PDF) [file pntd.0012883.s001.pdf]

Correct Installation Placement

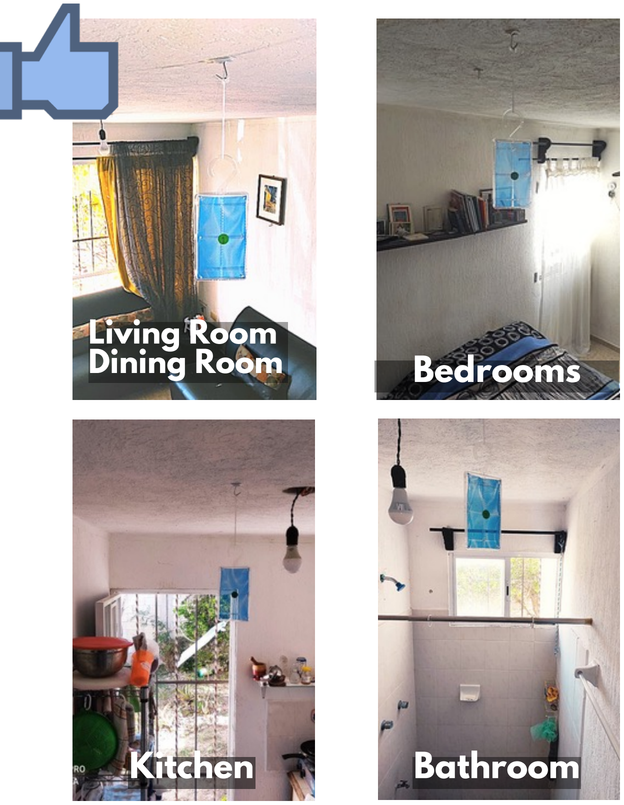

Incorrect Installation Placement

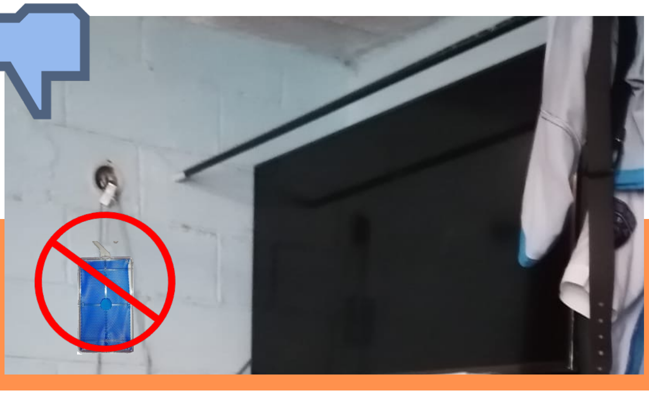

- **Place** the emanator as close to the center of the room as possible. **Do not hang** the emanator near a window or against a wall.
- **Avoid** placing emanators in open areas or unused rooms (e.g., patios or storage areas).
- As a **precaution**, **do not** hang the emanator from electrical wires.

To ensure the emanators are working effectively, our team will visit your home every three weeks to check for mosquito reduction.

CONTACT US

[illegible]

[illegible]

Designated space for contact information (phone numbers, email, social media, website)

This product is provided free of charge by the Collaborative Unit for Entomological Bioassays (UCBE - UADY).

Space designated for the logos of the UCBE/UADY, Emory University and Queensland Institute of Medical Research

Project Emanators

Space designated for the logo of Autonomous University of Yucatán (UADY)

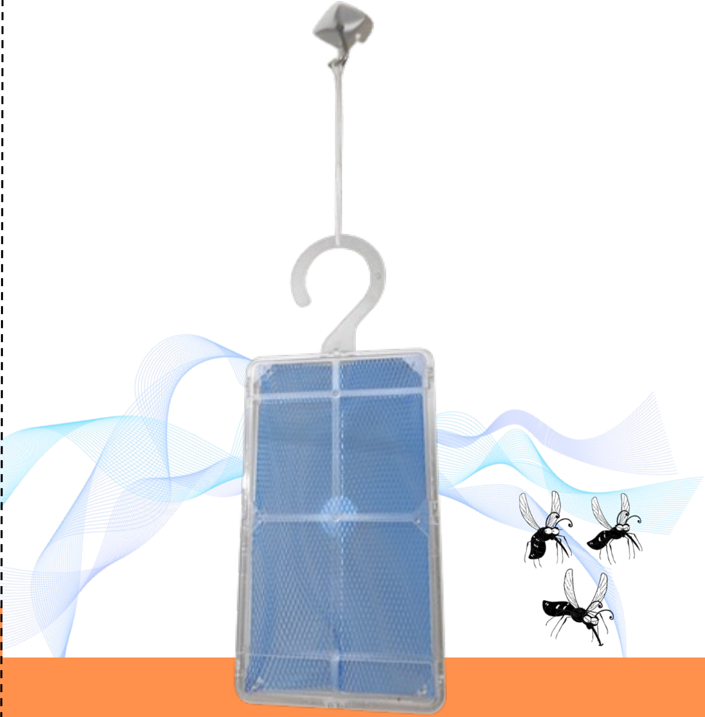

HOME MOSQUITO REPELLENT

- Easy to install
- Effective for up to 3 weeks
- Free and safe

EMANATORS

Emanators are designed to passively disperse a non-toxic insecticide, providing effective mosquito bite prevention inside your home.

They consist of a **plastic mesh** infused with **metofluthrin**, a volatile insecticide developed by Sumitomo Chemical Company Ltd. (Chuo-ku, Tokyo, Japan). Unlike traditional mosquito coils, emanators repel mosquitoes without producing smoke or requiring electrical heating, as plug-in devices do.

The insecticide-impregnated mesh is enclosed in a plastic frame tailored for indoor use. It can be hung in areas such as kitchens, bedrooms, living rooms, or dining spaces, where gentle air circulation promotes the gradual release of the insecticide.

Previous studies have demonstrated that emanators are highly effective against the **mosquito *Aedes aegypti***, the primary mosquito vector for diseases such as dengue, Zika, and chikungunya.

RECOMMENDATIONS

- Emanators remain effective for up to 3 weeks once removed from their plastic packaging. It is recommended to replace the emanator every 3 weeks.
- Hang the emanator from the ceiling to maximize airflow and ensure the steady release of the insecticide.
- Use one emanator per occupied room or every 3–4 meters.
- Emanators are safe for children and pets; however, avoid direct contact or ingestion.
- You will receive multiple packages for installation on specific dates. Store unused emanators in a drawer until they are ready for use.
- Dispose of used emanators in non-recyclable trash.

INSTALLATION PACKAGE

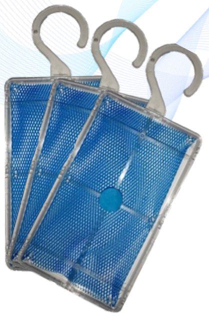

Interchangeable emanators: Four packages are included, each lasting three weeks, providing a total of 12 weeks of protection.

Rubber installer: Adjustable to fit any broomstick.

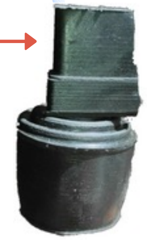

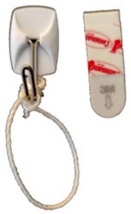

Hooks with self-adhesive tape: Designed for secure ceiling attachment.

INSTALLATION INSTRUCTIONS

1. Attach the rubber installer to a broomstick and peel off the protective backing from the self-adhesive hooks. Stick the hooks onto the installer.

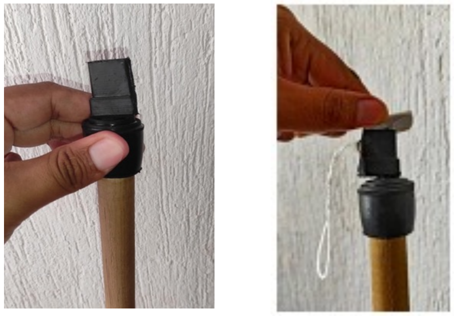

2. Once the hook is affixed to the rubber installer, press the hook firmly against the ceiling to attach it securely.

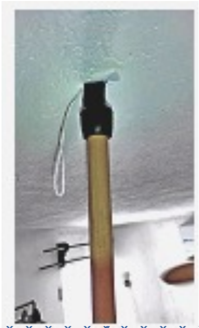

3. Take the emanator from the package labeled for the current installation date. Remove the plastic wrapping and place the date label (included in the package) in the center of the emanator.

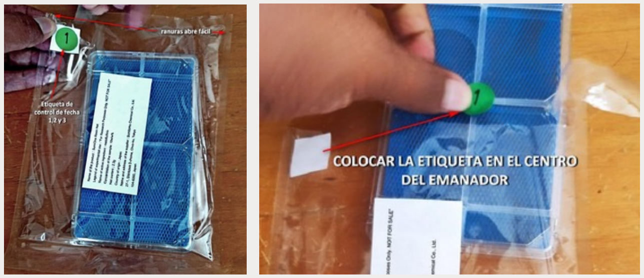

4. Clip the plastic hook on the emanator into the corresponding slots on the frame.

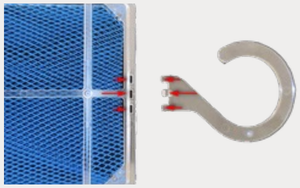

5. Use the rubber accessory to hang the emanator from the thread attached to the ceiling hook.

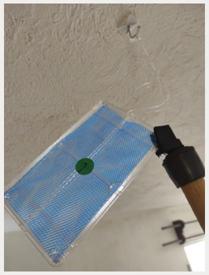

6. Repeat steps 3 to 5 on the indicated dates for each package. The packages are color-coded to help you keep track of the schedule.

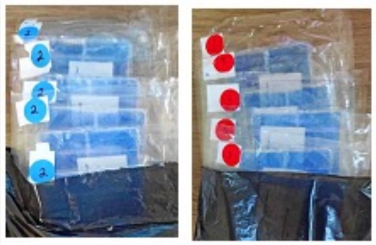

7. When the three-week period is over, dispose of the used emanator in non-recyclable trash.
