# Supplementary material for: Community deployment of metofluthrin emanators to control indoor Aedes aegypti: Efficacy results from a crossover trial in Yucatan, Mexico
Source: PLoS Negl Trop Dis. 2025 Sep 5;19(9):e0012883. doi: 10.1371/journal.pntd.0012883 (PMC12422581; doi:10.1371/journal.pntd.0012883)
Supplement: S1 Table — (DOCX) [file pntd.0012883.s002.docx]

S2 Table. Overall user satisfaction by implementation model before and after the crossover.

| **Fase** | **Implementation model** | **Not satisfied** | **Somewhat satisfied** | **Very satisfied** | **Total** |
| --- | --- | --- | --- | --- | --- |
| Before | MD | 0 (0) | 7 (3.6%) | 187 (96.4%) | 194 |
|  | CD | 1 (0.5%) | 8 (4.1%) | 185 (95.4%) | 194 |
| After | CD | 1 (0.5%) | 8 (4.2%) | 180 (95.2%) | 189 |
|  | MD | 1 (0.6%) | 6 (3.5%) | 165 (95.9%) | 172 |
