# Supplementary material for: Community deployment of metofluthrin emanators to control indoor Aedes aegypti: Efficacy results from a crossover trial in Yucatan, Mexico
Source: PLoS Negl Trop Dis. 2025 Sep 5;19(9):e0012883. doi: 10.1371/journal.pntd.0012883 (PMC12422581; doi:10.1371/journal.pntd.0012883)
Supplement: Attachment — Submitted filename: PNTD-D-25-00136_Review_Comments.docx [file pntd.0012883.s003.docx]

Reviewer Comments for “Community deployment of metofluthrin emanators to control indoor *Aedes aegypti*: efficacy results from a crossover trial in Yucatan, Mexico.”:

This work is direct, concise, and strong. This is a fantastic paper that is laid out clearly and has a valuable message for scalability of an alternative outbreak response paradigm.

**Key Words:**

All of your key words are redundant with your title. Consider other options such as intervention, mass deployment, vector management, spatial repellent, pyrethroid, community behavior, bite prevention, etc.

**Author Summary:**

Line 72: Vectors do not transmit diseases. They transmit pathogens, the causal agents of disease.

After reading the rest of the paper, I think you should have in both the abstract and author summary a pointed statement that these were highly pyrethroid resistant mosquito populations and the intervention was significant in spite of it.

**Introduction:**

Line 89: the common name is the yellow fever mosquito.

Line 90: These citations aren’t really about these aspects of their biology. Endophilic is acceptable as a descriptor, though consider saying anthropophilic to be more cautious of the wildly varying and cryptic larval sources they utilize all over the world. I would not describe them as endophagic; they don’t prefer to bite indoors at the exclusion of other environments. They are opportunistic, biting at many times of day and wherever you happen to encounter them.

**Materials/Methods:**

Line 219-222: Were there any source reduction/environmental control/hygiene steps as part of the routine vector control? Many countries start this first and continue it throughout seasonal and outbreak management.

Were there any ethics review documents you should proffer for the human landing catches or for volunteer participation in trials?

**Results:**

Fig 2: Any thoughts about the up and down nature of the numbers in spite of treatment? Is this the treatment wearing off and not quite lasting to the end of the designed interval?

Table 3: Excellent. Data really can’t be clearer than that for this type of study.

Line 415-419: Oof. That’s unfortunate. But it is good to see the repellency function is clearly intact, as has been suggested by lab and field evaluations of repellent function. I am really glad you pursued this. It is a tremendously important message that these intervention works in spite of the resistance. The 30% efficacy is much easier to swallow when you consider this is an otherwise refractory group of mosquitoes.

**Discussion:**

Line 436-438: You should accentuate the resistance argument more. Did those prior studies also monitor their resistance and show the 30%+ effect despite the resistance? If so, make that clear that this is consistently working in resistant populations. If not, then highlight that your study took that extra step and it may infer that those other successful trials also experienced severe resistance.

Line 458-459: Wonderful! Implementation-friendly. It is rare that a tool this compatible with untrained users makes its way into vector control.
