# Supplementary material for: Community deployment of metofluthrin emanators to control indoor Aedes aegypti: Efficacy results from a crossover trial in Yucatan, Mexico
Source: PLoS Negl Trop Dis. 2025 Sep 5;19(9):e0012883. doi: 10.1371/journal.pntd.0012883 (PMC12422581; doi:10.1371/journal.pntd.0012883)
Supplement: Attachment — Submitted filename: Response to Reviewers.docx [file pntd.0012883.s005.docx]

**RESPONSE Manuscript ID PNTD-D-25-00136**

**PLOS Neglected Tropical Diseases**

**Dear Editor**

We appreciate very much the review on our manuscript titled “**Community deployment of metofluthrin emanators to control indoor *Aedes aegypti*: efficacy results from a crossover trial in Yucatan, Mexico**". We have taken them all into consideration (including the journal requirements:) and provide below a point-by-point response to each one of them. Our response is marked on bold font, and starts with a letter R.

I confirm that I am the photographer of Figure S1. Regarding the clip-art images in the figure panels, the images of the thumbs up/down hands, the pointing hand, and the mosquitoes have been replaced with open-source resources (https://openclipart.org). The project logo design (including the image of the emanator) is my own work. The logos of the universities and institutions that participated in the project have been removed and replaced with a descriptive caption. Finally, the image containing an identifiable person has been replaced, and all personal data (names and phone numbers) of study participants have been removed and replaced with a blurred image. For Figure 1, the appropriate attribution has been added to the figure legend.

I confirm that all authors have seen and approved the final text.

On behalf of my co-authors.

**REVIEWER(S)' COMMENTS TO AUTHOR:**

**Response to the reviewers' comments:**

**Methods**

Reviewer #1: Were there any source reduction/environmental control/hygiene steps as part of the routine vector control? Many countries start this first and continue it throughout seasonal and outbreak management.

**R: Although the Ministry of Health carries out cleanup campaigns (known as *descacharrización*) once or twice a year (since 2012) to eliminate potential *Aedes* breeding sites—and the year the study was conducted was no exception—these efforts are limited to the city of Mérida and its surrounding districts of this municipality. Our study sites, located in the suburban municipality of Umán, were not included in these activities.**

Were there any ethics review documents you should proffer for the human landing catches or for volunteer participation in trials?

**R: Information regarding the study's ethical approval has been added; an 'Ethics Statement' section was included in the 'Materials and Methods' section, as follow: *This study was approved by the Ethics and Research Committee of the Universidad Autónoma de Yucatán (Approval No. UPI/393/2021). Written informed consent was obtained from all participants (heads of households) and each volunteer (field technician).***

Reviewer #2: Keys for identification of the mosquitoes are missing

**R= Thank you for the comment. We mentioned in the methods that mosquito identification was carried out by expert personnel familiar with the identification of *Aedes aegypti* adults.**

Reviewer #3: 1. It should be stated explicitly in the Methods and the abstract that the trial design was non-blinded/open-label

**R= Many thanks, we included this suggestion in the Abstract and Methods.**

2. The description of the units of randomisation and treatment assignment at lines 169-177 on p10 is not clear enough in several aspects:

2a. This sentence ‘Clusters were defined as entire city blocks randomly selected from suburban blocks within the three neighborhoods of study’ is confusing. The distinction between ‘city blocks’ and ‘suburban blocks’ is not clear, nor is it clear how the unit of ‘block’ differs from ‘cluster’. Please state how many eligible city blocks there were in total from which this subset of 42 clusters was selected and what method was used for random selection of those 42 clusters for inclusion in the trial - e.g. probability proportionate to size? Was the selection stratified by the 3 neighbourhoods?

2b. Among the 42 clusters, it looks from Fig 1 like assignment to the three study arms was randomised within each of the 3 neighborhoods because the cluster numbers per arm are exactly balanced within each neighborhood - if so this stratified randomisation needs to be stated in the methods.

2c. The unit of randomization is the cluster, but the unit of intervention is the household and it is not clear what proportion of total houses in each cluster (and overall in each study arm) received a spatial emanator. Line 175 states “For treatment clusters, the goal was to achieve full block coverage with SE.” Does this mean the goal was to deploy an SE in every household in each intervention cluster, or if not what does ‘full block coverage’ mean? It is stated further on (p13) that each cluster included 14 participating houses - please include this information with the description of treatment assignment on page 10, and state the total number of houses in each cluster and arm and how the participating 14 houses were selected.

**R: Thank you for these helpful comments. We have clarified the description of randomisation and treatment assignment in the revised manuscript as follows:**

**2a. We have revised the text to improve clarity. The term “city blocks” and “suburban blocks” were used interchangeably and imprecisely. To clarify the sentence: *Clusters were defined as entire blocks of houses, and all clusters were selected from suburban areas within the three neighborhoods included in the study. From a total of 173 eligible blocks identified across the three neighborhoods, 42 blocks (clusters) were randomly selected using simple random sampling stratified by neighborhood, to ensure balanced allocation among arms and neighborhoods.***

**2b. As correctly noted, randomisation was stratified by neighborhood. Within each neighborhood, 14 clusters were selected and randomly allocated to one of the three study arms (i.e., 4–5 clusters per arm per neighborhood), resulting in a balanced distribution across the trial (see updated Fig 1). This stratified randomisation is now clearly described in the “Study design” section.**

**2c. The unit of randomisation was the cluster (i.e., block), while the unit of intervention and data collection was the household. Each cluster included 14 participating households (households required to meet the study's sample size), which were selected from the total number of households in the block. For treatment clusters, the aim was to achieve “full block coverage,” which we now clarify to mean that one spatial emanator (SE) was deployed in each of the 14 participating households. We did not attempt to treat all households in the block, but instead focused on consistent and complete coverage within the 14 enrolled homes per cluster. This clarification has been added to the description of the intervention on page 10 to ensure transparency regarding the proportion of houses treated.**

**We have updated the Methods section accordingly to clarify the definitions and procedures used.**

3. Regarding the Entomological endpoints (page 14):

3a. Please state which were the pre-defined primary and secondary endpoints for evaluating entomological efficacy: total Ae. aegypti; female; blood-fed? And report results for all of the primary and secondary endpoints (see comment 2 re Results).

3b. Why was entomological sampling only done in 50% of houses? This means that the effective sample size of the trial for the purpose of the analyses was only 7 houses per cluster x 14 clusters, and not 14 houses x 14 clusters as stated in the power calculations - as the remaining half of the houses contributed no information on entomological outcomes, if I understand the methods correctly.

3c. Please clarify whether entomological sampling was conducted ‘once a week following each cycle of installation’ as stated at line 267 (i.e. 24 sampling weeks) or whether this should read ‘one week’ (i.e. 8 sampling weeks, as suggested by Figure 2.

3d. I have particular concerns about the analyses of human landing catch data as a secondary endpoint, as these data were only collected in a total of 12 houses (4 per arm), repeated at 4 timepoints. No information is provided about how those 4 houses per arm (of 196 total per arm) were selected, and as such it is not possible to draw robust conclusions from these data.

**R: Thank you for the comments. We have clarified the description of entomological points as follow:**

**3a. Our trial used households as the units of treatment and analysis, and assessed the entomological impacts of continuous (cyclical) SE deployment using the indoor Aedes aegypti adult abundance (including female abundance, blood-fed female abundance) as primary endpoint, and estimates of *Ae. aegypti* landing behavior, as a secondary endpoint.**

**3b. Your observation is correct — entomological sampling was conducted in 50% of the participating households. Although entomological sampling was conducted in only 50% of the participating households, the study design maintained sufficient statistical power to detect meaningful differences in mosquito abundance. In our previous trial (e.g., Devine GJ et al. [2021] The entomological impact of passive metofluthrin emanators against indoor Aedes aegypti: A randomized field trial. PLoS Negl Trop Dis 15(1): e0009036), a two-arm randomized controlled trial with 100 houses per arm, we achieved adequate power to detect a 60% reduction in abundance rate ratios. Therefore, sampling 50% of houses per cluster (7 out of 14) still provided adequate power for the intended entomological comparisons within the crossover design.**

**3c. One entomological survey was conducted per deployment cycle. So should read *one week (i.e. 8 sampling weeks)*.**

**3d. The selection of the four households per arm for human landing catch (HLC) was based on resident willingness and ease of access. This method is more intrusive than aspirator-based adult mosquito collections, as it requires three field technicians to enter the home, conduct a 10-minute evaluation, and prepare the setup in advance. Only households that were fully informed of the HLC procedure and agreed to participate in four assessments throughout the study period were included. Not all households agreed to participate or provided consent. In previous studies (e.g., Devine GJ et al. [2021] The entomological impact of passive metofluthrin emanators against indoor Aedes aegypti: A randomized field trial. PLoS Negl Trop Dis 15(1): e0009036), we have used a similar number of HLC houses, acknowledging the difficulty in obtaining consent and the increased exposure risk to field staff.**

**We have updated the Entomological Endpoint section accordingly to clarify the definitions and procedures used.**

4. Regarding the description of the GLMM statistical analysis (page 16)

4a. The terminology regarding clusters (level 1) and block (level 2) is confusing. What does a block correspond to here? Clustering in entomological indices at the household level should also be accounted for in the GLMM analysis, since the mosquito sampling was repeated over time at the same households and those repeated observations are not statistically independent.

4b. In fact, the text in the Results section indicates that models did include House ID as a random effect, but this is at odds with the model described at lines 311-2 of Methods. Please clarify and strengthen the description of the statistical modelling methods used.

**R: Many thanks, the comment is very accurate. Indeed, the terminology was confusing and contained an error. The nesting was at the cluster level (level 1) and household level (level 2). We have corrected and clarified this appropriately in the analysis section.**

5. No ethical considerations are included in the methods section, regarding review and approval of the study protocol by an IRB or regarding informed consent obtained from trial participants. This information must be included

**R: Information regarding the study's ethical approval has been added; an 'Ethics Statement' section was included in the 'Materials and Methods' section, as follow: *This study was approved by the Ethics and Research Committee of the Universidad Autónoma de Yucatán (Approval No. UPI/393/2021). Written informed consent was obtained from all participants (heads of households) and each volunteer (field technician).***

**Results**

Reviewer #1: Any thoughts about the up and down nature of the numbers in spite of treatment? Is this the treatment wearing off and not quite lasting to the end of the designed interval?

**R: Thank you for the comment. The study was conducted from July to December, coinciding with the rainy season (starting around May/June, peaking between July and September, and transitioning to the dry season in November). The fluctuations observed in Figure 2 likely reflect the natural seasonal dynamics of mosquito populations. The initial declines in abundance during the first four deployment cycles (July–September) suggest a strong treatment effect. After the fifth cycle, the continued decline across all arms likely corresponds to the natural population drop as the dry season began. Rather than a loss of treatment effect, we believe this pattern reflects the overall decrease in *Ae. aegypti* population abundance, which in turn leads to less pronounced reductions in entomological indices. This phenomenon has also been observed in our previous intervention studies conducted during the same seasonal window in the region (Vazquez-Prokopec GM, et al. (2017). PLoS Negl Trop Dis 11(6): e0005656; Manrique-Saide P et al. (2021). PLoS Negl Trop Dis 15(1): e0009005; Vazquez-Prokopec GM, et al. (2022). Sci Rep. 2022 Dec 20;12(1):21998) , where detecting statistically significant differences between study arms became increasingly difficult when mosquito densities were low.**

Otherwise, this is really excellent. Data really can’t be clearer than that for this type of study.

**R: Thank you! We’re glad to hear that the data presentation in Table 3 was clear and effective.**

Reviewer #2: The data is well analyzed.

**R: Thank you!**

Reviewer #3: 1. The description of the crossover analysis at lines 356-60 belongs in the methods section and, as above, is inconsistent with the description of the statistical model at lines 311-2 of Methods.

**R: Thank you for the observation. The description of the crossover analysis has been moved to the data analysis section, along with the clarifications already made regarding the model description.**

2. Figure 2 shows results for number of total Aegypti and number of blood-fed females per house, while the results of statistical models are only reported for the total Aegypti endpoint and not blood-fed females; as per my comment on the Methods, please report results for each of the pre-defined primary and secondary endpoints.

**R: Thank you for your observation. While blood-fed female abundance was included as a predefined primary endpoint and is shown descriptively in Figure 2, the statistical models focused on total adult Ae. aegypti abundance because this variable showed greater stability and model fit across time points and arms. In contrast, blood-fed female counts were often low and more variable, which led to convergence issues and reduced model reliability. We have clarified this point in the Results sections, and we now explicitly state that model-based estimates were only presented for endpoints with adequate data distribution and model performance.**

3. In reporting the intervention effect on p17, the authors state “...with this effect lasting until the fourth survey date”, but Figure 2 shows no difference between CD or MD groups vs Control in the Post 4 survey.

**R: Thank you for the insightful observation. The text has been corrected accordingly—“fourth survey” was replaced with “third survey” to accurately reflect that the strongest intervention effect was observed up to that survey point, as shown in Figure 2.**

4. In addition to my comment on the Methods for selecting houses for human landing catches, it is not clear from the data presented in Table 4, and the statistical methods described at lines 321-24, how the efficacy of the intervention was estimated from the human landing catch data.

**R: Thank you for your helpful comment. We now clarify how efficacy was estimated from the human landing catch (HLC) data. As stated in the Data Analysis section, we used a generalized linear mixed model (GLMM) with a Poisson link function and a random effect for House ID to account for repeated measures within houses. Efficacy was calculated using the incidence risk ratio (IRR) derived from the model outputs for each time point, applying the formula: Efficacy = 1 − IRR. For example, at Post 4, the model estimated IRRs of 0.21 for CD and 0.14 for MD, corresponding to predicted reductions in landing rates of 79% and 86%, respectively (Table 4). We have updated the Results section to reflect these calculations and ensure consistency between the statistical methods and how intervention efficacy is reported.**

4a. Please report the absolute HLC data (e.g. mean/median and range of attempted landings per house and per time point) as well as the model output shown in Table 4

**R: Done. We included a column with estimated HLC with its respective 95% CI.**

4b. Clustering of observations at the household level should be accounted for in this analysis - was it?

**R: Yes, clustering of observations at the household level was accounted for in the analysis. Human landing catch data were analyzed using a generalized linear mixed model (GLMM) with a Poisson link function, and a random effect was included for each household (House ID). This approach addressed the repeated measurements taken over time within the same households and ensured that the lack of independence among observations was properly modeled. This clarification has been added to the statistical methods section for transparency.**

4c. The IRR value for the reference group (Control arm) would usually be 1.0; I don’t understand the values of IRR reported for the ‘intercept’ arm in Table 4.

**R: Thank you for the observation. The necessary modifications have been made to the table 4 to improve clarity.**

**Conclusions**

Reviewer #1: You should accentuate the resistance argument more. Did those prior studies also monitor their resistance and show the 30%+ effect despite the resistance? If so, make that clear that this is consistently working in resistant populations. If not, then highlight that your study took that extra step and it may infer that those other successful trials also experienced severe resistance.

**R=Previous studies—including one conducted by our research group (e.g., Devine GJ et al. [2021] The entomological impact of passive metofluthrin emanators against indoor Aedes aegypti: A randomized field trial. PLoS Negl Trop Dis 15(1): e0009036)—have monitored resistance and still reported effects greater than 30% despite it. Following the reviewer’s suggestion, we have revised the paragraph to highlight more clearly that this intervention has consistently demonstrated efficacy in resistant mosquito populations. The revised text now reads: *Entomological impact was consistent with other previously reported results (12,13), adding more evidence and confirming that emanators and/or spatial repellent formulations like these, can have an important impact on Ae. aegypti population densities and human-vector contact indoors. Importantly, this intervention has consistently demonstrated efficacy in resistant mosquito population (12), reinforcing its potential utility for public health vector control responses against ABVs.***

It is rare that a tool this compatible with untrained users makes its way into vector control.

**R= Thank you! We agree that the ease of implementation by untrained users is a key strength of this intervention.**

Reviewer #2: satisfactory

**R= Thank you!**

Reviewer #3: 1. The statement in the first line of the discussion that the intervention resulted in significant reductions in Ae. aegypti females/blood-fed females and the number of attempted landings is not supported by the data presented

1a. As per comments above, data in Figure 2 shows only total Ae. aegypti and blood-fed females, not Ae. aegypti female counts. And the statistical model results presented only include total Ae. aegypti, not females or blood-fed females. Results must be shown for pre-defined endpoints.

**R: The sentence has been revised to refer exclusively to total adult Aedes aegypti mosquitoes, in alignment with the data presented and the statistical model results.**

1b. The very small number of houses in which HLC data was collected; the lack of explanation of how those houses were selected; the lack of reporting of the raw data on HLC results; and the apparent lack of accounting in the statistical analysis for the clustering of data within (the very small number of) households together make the HLC very unconvincing. The above details should be reported, and the conclusions drawn from these data should be tempered to explicitly acknowledge the limitations of small sample size and (presumably) large inter- and intra-household variability in attempted landings

**R: In the discussion (after first paragraph) was added the following: *Although human landing catch (HLC) data were collected from a small number of houses (n=4 per arm), this approach followed established protocols from previous ES studies (12) and was chosen for logistical and ethical reasons. Repeated measures within these households over time allowed for consistent assessment of host-seeking behavior. Despite the limited sample size, the observed reductions in mosquito landings align with trends in adult mosquito abundance, providing important complementary evidence that metofluthrin emanators reduce human-vector contact indoors.***

2. ‘Emanator status’ is referred to in the discussion, but there is no mention in the methods section about how, when or by whom placement accuracy was assessed.

3. The authors introduce for the first time in the discussion section (lines 472-84) the information that an acceptability/satisfaction survey was conducted among trial participants to understand community preferences. Please include the acceptability survey methods and results in the paper; this cannot be introduced in the discussion section without any data shown. Given that the study was focused on evaluating community deployment of spatial emanators, it seems very odd to have excluded this qualitative data from the paper.

**R: We have added the details regarding emanator status and the acceptability/satisfaction survey to the Methods and Results sections. This includes how, when, and by whom the placement accuracy was assessed, as well as the survey methods and findings related to community preferences, ensuring these important aspects are fully integrated into the paper.**

**Editorial and Data Presentation Modifications?**

Reviewer #1: Minor revision

**R= Thank you!**

Reviewer #2: Minor revision

**R= Thank you!**

Reviewer #3: Minor comments:

1. Line 110: spell out the abbreviation ‘a.i. w/w’ at first use

**R: Done.**

2. Lines 219-222: specify whether the routine vector control activities also continued in the intervention arms.

**R: We specified that routine vector control activities also applied to the intervention arm.**

**Summary and General Comments**

Reviewer #1: This work is direct, concise, and strong. This is a fantastic paper that is laid out clearly and has a valuable message for scalability of an alternative outbreak response paradigm.

**R: We thank the reviewer for the constructive opinions and feedback on our manuscript.**

Just a couple things:

Key Words:
All of your key words are redundant with your title. Consider other options such as intervention, mass deployment, vector management, spatial repellent, pyrethroid, community behavior, bite prevention, etc.

**R: Many thanks, we considered other option as suggested: *Spatial emanator, community behaviour, mass deployment*.**

Author Summary:
Line 72: Vectors do not transmit diseases. They transmit pathogens, the causal agents of disease.

**R: Many thanks, the comment is very accurate. We rewrote the sentence as: *Spatial emanators are an innovative and user-friendly tool for controlling Aedes mosquitoes that transmit viruses such as dengue, chikungunya, and Zika.***

After reading the rest of the paper, I think you should have in both the abstract and author summary a pointed statement that these were highly pyrethroid resistant mosquito populations and the intervention was significant in spite of it.

**R: Many thanks, we included this suggestion in the abstract and author summary.**

Introduction:
Line 89: the common name is the yellow fever mosquito.

**R: Many thanks, rewritten as suggested.**

Line 90: These citations aren’t really about these aspects of their biology. Endophilic is acceptable as a descriptor, though consider saying anthropophilic to be more cautious of the wildly varying and cryptic larval sources they utilize all over the world. I would not describe them as endophagic; they don’t prefer to bite indoors at the exclusion of other environments. They are opportunistic, biting at many times of day and wherever you happen to encounter them.

**R: We rewrote this sentence, as follow: *The yellow fever mosquito Aedes aegypti, the primary urban vector of dengue, Zika, and chikungunya viruses, is an opportunistic species that is highly, if not exclusively, anthropophilic, feeding preferentially on humans***.

Reviewer #2: It is an interesting study. However, I have three concerns: (1) The identification of the mosquito species is not presented. (2) The human landing catch method for Aedes aegypti requires ethical approval, but this is not mentioned. (3) The year and date of the study are missing.

**R: We thank the reviewer for the constructive opinions and feedback on our manuscript.**

**(1) At the beginning of the results section, the identified mosquito species are specified.**

**(2) Information regarding the study's ethical approval has been added; an 'Ethics Statement' section was included in the 'Materials and Methods' section, as follow: This study was approved by the Ethics and Research Committee of the Universidad Autónoma de Yucatán (Approval No. UPI/393/2021). Written informed consent was obtained from all participants (heads of households) and each volunteer (field technician).**

**(3) The study was conducted from July to December 2021, coinciding with the rainy season (starting around May/June, peaking between July and September, and transitioning to the dry season in November). We have updated the Methods section accordingly to clarify this.**

Reviewer #3: The authors report the outcomes of a three arm cluster randomized controlled trial of the entomological efficacy of metofluthrin spatial emanators in reducing indoor Ae. aegypti abundance and biting behavior in a Mexican city. The study was designed specifically to test the hypothesis that entomological efficacy is comparable when the emanators are installed by community members with limited instruction, vs the managed approach to deployments that has been used in the previous trials that have reported entomological efficacy of this intervention. As outlined in my specific comments above, I have considerable concerns with the lack of clarity in describing the approach used for cluster selection and treatment assignment; the incomplete reporting of all entomological outcomes; and the fact that the effective sample size was half of what is stated in the methods because the entomological endpoints appear to have been measured in only 50% of households enrolled in the trial; and with the sampling approach, analysis and reporting of the HLC data, all of which need to be addressed in a revised manuscript.

**R: Thank you very much for your valuable comments; they have undoubtedly improved the clarity of the manuscript's results. We hope we have addressed each of your concerns adequately.**
