# Supplementary material for: Community deployment of metofluthrin emanators to control indoor Aedes aegypti: Efficacy results from a crossover trial in Yucatan, Mexico
Source: PLoS Negl Trop Dis. 2025 Sep 5;19(9):e0012883. doi: 10.1371/journal.pntd.0012883 (PMC12422581; doi:10.1371/journal.pntd.0012883)
Supplement: Attachment — Submitted filename: Response to Reviewers2.docx [file pntd.0012883.s006.docx]

August 12, 2025

**RESPONSE Manuscript ID PNTD-D-25-00136**

**PLOS Neglected Tropical Diseases**

**Dear Editor**

We greatly appreciate this second round of observations on our manuscript titled “**Community deployment of metofluthrin emanators to control indoor Aedes aegypti: efficacy results from a crossover trial in Yucatan, Mexico**.” We have carefully considered all comments and provide below a point-by-point response to each one. Our response is marked on bold font, and starts with a letter R.

We confirm that all figures meet PLOS requirements, as verified using the Preflight Analysis and Conversion Engine (PACE) digital diagnostic tool.

On behalf of my co-authors.

**REVIEWER'S COMMENTS:**

**Response to the reviewers' comments:**

**Methods**

*-Are the objectives of the study clearly articulated with a clear testable hypothesis stated?*

*-Is the study design appropriate to address the stated objectives?*

*-Is the population clearly described and appropriate for the hypothesis being tested?*

*-Is the sample size sufficient to ensure adequate power to address the hypothesis being tested?*

*-Were correct statistical analysis used to support conclusions?*

*-Are there concerns about ethical or regulatory requirements being met?*

Reviewer #2: ok.

**R: Thank you!**

Reviewer #3: The response regarding the effective sample size of the trial is not sufficient. Since entomological sampling was conducted in only 50% of households, then the effective sample size for measurement of the endpoints is 7 households x 14 clusters per arm. The additional 7 households per arm in which emanators were installed provide no information on the endpoints, therefore do not contribute to the sample size for the purpose of statistical power.

**R: Many thanks for this accurate observation. We have recalculated the statistical power considering 7 households (per cluster for each entomological survey date):**

***Starting from your observed powers with m=14 and scaling by √r:***

***MD vs Control: old power 0.99 .***

***When we re-run 100 Monte Carlo sims, we expect 95% CI ≈ 0.89–0.98.***

***CD vs Control (35% efficacy assumption): old power 0.83.***

***With 100 sims we’d expect 95% CI ≈ 0.60–0.79.***

**These results are within the 60% we established.**

This needs to be addressed by

i. adjusting the sentence at line 265-7 to read "we assessed the impact of metofluthrin emanators on the entomological endpoints in approximately 100 houses per treatment arm, i.e. 50% of the households in which emanators were installed."

**R: Done.**

and ii. Re-running the Monte Carlo simulations to reflect the actual trial design, in terms of the information generated on intervention effect (i.e. 14 clusters, each with 7 houses) and updated all of the power estimates reported in the 'Power and sample size calculations' section accordingly.

**R: Done.**

2. Thank you for the additional information regarding the human landing catches. Please updated the sentence at line 304 accordingly: "Human landing counts (12) were conducted in 12 houses (4 per arm) selected based on high baseline entomological indices, resident willingness and ease of access".

**R: Done.**

**Results**

*-Does the analysis presented match the analysis plan?*

*-Are the results clearly and completely presented?*

*-Are the figures (Tables, Images) of sufficient quality for clarity?*

Reviewer #2: ok

**R: Thank you!**

Reviewer #3: Yes

**R: Thank you!**

**Conclusions**

*-Are the conclusions supported by the data presented?*

*-Are the limitations of analysis clearly described?*

*-Do the authors discuss how these data can be helpful to advance our understanding of the topic under study?*

*-Is public health relevance addressed?*

Reviewer #2: ok

**R: Thank you!**

Reviewer #3: Yes

**R: Thank you!**

**Editorial and Data Presentation Modifications?**

*Use this section for editorial suggestions as well as relatively minor modifications of existing data that would enhance clarity. If the only modifications needed are minor and/or editorial, you may wish to recommend “Minor Revision” or “Accept”.*

Reviewer #2: ok

**R: Thank you!**

Reviewer #3: (No Response)

**R: Thank you!**

**Summary and General Comments**

*Use this section to provide overall comments, discuss strengths/weaknesses of the study, novelty, significance, general execution and scholarship. You may also include additional comments for the author, including concerns about dual publication, research ethics, or publication ethics. If requesting major revision, please articulate the new experiments that are needed.*

Reviewer #2: The revision is satisfactory.

**R: Thank you!**

Reviewer #3: The revised manuscript is much improved, and the authors have addressed the majority of my previous comments with the exception of two issues in the Methods section which still need to be addressed in a subsequent revision.

**R: Thank you!**
